# On the Conjoint Exhibition of the Eupatorium Perfoliatum, and Super-Tartrate of Potassa, in Tinea Capitis

**Published:** 1839-03-30

**Authors:** William Zollickoffer

**Affiliations:** Middleburg, (Md.)


*On the conjoint exhibition of the Eupatorium Perfoliatum, and Super-Tartrate of Potassa, in Tinea Capitis.* By William Zollickoffer, M. D.

The combination under consideration, being productive of the effects of a *general alterative*, has decided advantage over the internal remedies and external applications commonly resorted to in the treatment of *Tinea Capitis*. It is more prompt and effectual, and seldom fails to remove the disease in a very short time. *Tinea Capitis* may be controlled in its progress, and radically cured in from five to six weeks, by its use, without the necessity of having recourse to the various depilatory applications recommended by authors, all of which are productive of unnecessary uneasiness to the patient, to say nothing of the disagreeable effects growing out of such a course of treatment.

Considering this malady as the development of a general deranged condition in the office of the skin, locating itself on the scalp and its *immediate* proximity, produced by a degree of hepatic functional derangement, I have long since been persuaded that an alterative course of treatment is essential for its removal: and the practice I have adopted, based on this pathological view, has proved successful, whether this opinion of the doctrine of the diseased action constituting this troublesome malady be correct, or not. Preferring to the mercurial preparations, any remedy that is competent to the production of the same consecutive displays in the treatment of disease, in the affection which is the subject of this paper, I have found the combination recommended so efficacious, that I venture to solicit the profession to make a trial of its powers.

In using the perfoliatum, (the powdered leaves,) and cremor tartar, in the dose of ten grains of the former, with twenty of the latter, three times a day, from four to six weeks, desquamation will readily be found to take place, and the scalp to assume a healthy condition. I occasionally prescribe, particularly if the bowels are constipated, a dose of the tartras sodæ et potassæ, or sodæ sulphas: and, if the subject of the disease is of a plethoric habit, the dietetic course is strictly antiphlogistic: the latter precautionary means should be carried out to a certain extent under all circumstances. External applications, when this remedy is used, are altogether unnecessary. I exhibit the medicine in water, and occasionally add some sugar to render it more palatable.

*Middleburg*, (*Md.*) March, 1839.

